# Out-of-pocket expenditure among patients receiving plastic surgery care at an emergency department: A cross-sectional study

**DOI:** 10.6026/973206300213632

**Published:** 2025-10-31

**Authors:** Surya Senthil, Sailesh I.S Kumar, Madhumitha Palaniappan, Balaji Arumugam, Shoraf Pascal, Bhoovanachandaran M, Shanmukha Koppolu

**Affiliations:** 1 Department of General Medicine, Saveetha Medical College, Saveetha Institute of Medical and Technical Sciences, Thandalam, Tamil Nadu, India; 2 Department of Surgery, Madras Medical College, Chennai, Tamil Nadu, India; 3 Department of Pediatrics, Madha Medical college and research hospital, Tamil Nadu, India; 4 Department of Community Medicine, Arunai Medical College and Hospital, Tamil Nadu, India; 5 Department of Community Medicine, Madha Medical College, Kovur, Chennai, Tamil Nadu, India; 6 Department of Community Medicine, St Peter's Medical College Hospital and Research Institute, Tamil Nadu, India; 7 Department of Trauma and Orthopaedics, Barts NHS Trust, London, England, United Kingdom

**Keywords:** Financial strain, emergency room visits, plastic surgery, out-of-pocket expenses, medical bills and cross-sectional research

## Abstract

The out-of-pocket (OOP) expenses of 132 patients who sought plastic surgery care in an emergency department (ED) were evaluated. Financial stress was reported by a significant portion of patients due to the costs of urgent procedures, prescription medications and post-discharge care. Uninsured or underinsured patients have to deal with a much higher financial burden. The study highlights how better insurance coverage and cost transparency are necessary in emergency plastic surgery situations. Thus, we show the economic impact of acute treatment on patients' well-being.

## Background:

Plastic surgery interventions undertaken by emergency department staff are often aimed at addressing soft tissue injuries, hand trauma, facial lacerations and acute reconstructive procedures. Even though the surgical intervention is medically necessary, it can create considerable financial burden for the patient when performed in emergency settings that are costly, unwanted financial burdens [1]. In contrast to elective procedures, or other procedures requiring a patient to have typical non-emergency time to consider costs, urgent situations often do not allow a patient or provider any opportunity to plan for cost and therefore leave the patient with unwanted out-of-pocket (OOP) costs [2]. These OOP costs could include the surgeon/specialist bill, surgical procedure costs, anesthesia, imaging, surgical wound supplies and post-operative medications [3]. In countries with limited public or fractured health insurance systems, these OOP costs are often borne by the patient [4]. Even in systems with public or private insurance, there are often gaps in coverage, co-payments and services that are excluded in coverage leading to further financial burden for the patient [5]. Unplanned expenses for needed care create delays in care, ability to pay for services and eventually debt and often affect clinical outcomes as much as patient well-being [6]. Although plastic surgery services have become more commonplace in the Emergency Department (ED), to our knowledge, no studies have examined the financial implications from the patient's perspective [7]. Understanding the magnitude and factors related to out-of-pocket (OOP) costs is important in order to enhance discussions with patients, inform hospital and health system policies and interventions aimed to relieve the burden of these costs. Further, understanding OOP costs could lead to equity in healthcare by identifying vulnerable patients who may be subject to financial toxicity [8]. Therefore, it is of interest to report the extent of out-of-pocket costs incurred by patients undergoing urgent plastic surgery and to identify factors associated with increased financial burden.

## Materials and Methods:

Over the course of six months, this cross-sectional study was carried out in the emergency room of a tertiary care hospital that had a functioning plastic surgery unit. Following informed agreement, 132 patients who underwent plastic surgery for acute diseases, including soft tissue damage, hand injuries, face lacerations and modest reconstructive needs, were enrolled one after the other. Inclusion criteria comprised patients aged ≥18 years that required immediate or urgent plastic surgery consultation or intervention within the ED. Data were collected through face-to-face interviews using a pre-validated semi-structured questionnaire, administered within 72 hours of treatment. The questionnaire captured demographic details, type and extent of injury, nature of surgical intervention, insurance status and detailed components of out-of-pocket expenditure including registration charges, surgical fees, consumables, imaging, medications and follow-up visits. Over the course of six months, this cross-sectional study was carried out in the emergency room of a tertiary care hospital that had a functioning plastic surgery unit. Following informed agreement, 132 patients who underwent plastic surgery for acute diseases, including soft tissue damage, hand injuries, face lacerations and modest reconstructive needs, were enrolled one after the other. Patients with incomplete data, those unwilling to participate, or referrals from outside facilities were excluded. Descriptive and analytical statistics were applied to evaluate the patterns and predictors of financial burden.

## Results:

Out of 132 patients receiving emergency plastic surgery care, 58.3% were male, with a mean age of 34.6 years. Most patients (71.2%) reported out-of-pocket (OOP) expenditures, with higher costs significantly associated with lack of insurance, type of injury and the use of surgical consumables. Patients in the 21-40-year age range formed the majority and facial lacerations were the most frequent injury requiring intervention. Male patients slightly predominated, reflecting the demographic trend of injury presentations. The majority of patients lacked comprehensive insurance, resulting in direct payments for medical services. Medications and consumables were the highest contributors to OOP expenses. Total OOP expenditure was significantly higher in uninsured patients, who also accounted for the majority of high-burden cases (>INR 10,000). Patients undergoing surgical interventions incurred greater OOP costs compared to those managed conservatively. Multivariate analysis identified no insurance, surgical management and facial injury as significant predictors of high OOP burden. These findings underscore the financial vulnerability of patients requiring emergency plastic surgery and highlight the impact of insurance coverage on healthcare affordability.

Table 1 (see PDF) shows demographic profile of study participants depicts that most patients were aged 21-40 years, with a smaller proportion in younger (<20 years) and older (>50 years) age groups. Table 2 (see PDF) shows gender distribution of study population illustrates that males represented a slight majority (58.3%) of the study cohort. Table 3 (see PDF) shows type of injury requiring plastic surgical intervention highlights that facial lacerations were the most common injury, followed by hand trauma, soft tissue loss, nasal/oral injuries and burns. Table 4 (see PDF) shows insurance status of patients describes that more than half of the patients lacked insurance, while the remainder were covered under government schemes or private insurance. Table 5 (see PDF) shows proportion of patients reporting out-of-pocket expenditure presents that over 70% of patients reported some form of OOP spending, reflecting the financial burden of emergency care. Table 6 (see PDF) shows components of out-of-pocket expenditure depicts that medications and surgical consumables were the largest contributors to OOP costs, followed by surgical fees, imaging, registration and follow-up visits. Table 7 (see PDF) shows total mean out-of-pocket expenditure by insurance status illustrates that uninsured patients had the highest mean OOP expenditure (INR 10,870), with lower costs observed among those covered by government schemes or private insurance. Table 8 (see PDF) shows distribution of high OOP burden by insurance status highlights that uninsured patients accounted for the majority of high-burden cases (OOP > INR 10,000), compared to those with government or private insurance. Table 9 (see PDF) shows out-of-pocket expenditure by type of treatment compares OOP costs between patients undergoing surgical management and those managed conservatively, showing significantly higher costs in the surgical group. Table 10 shows multivariate logistic regression for predictors of high OOP expenditure indicates that lack of insurance, surgical management and facial injury were significant independent predictors of high OOP expenditure.

## Discussion:

This study highlights the substantial out-of-pocket (OOP) expenditure incurred by patients receiving emergency plastic surgery care, with over 70% of participants reporting financial payments despite seeking urgent treatment [9]. The economic burden was especially pronounced among uninsured individuals, who paid significantly more than those covered by government or private insurance. Consistent with previous findings in acute trauma and surgical care literature, consumables and medications were the primary cost drivers, often exceeding procedural or imaging expenses [10]. Patients undergoing surgical interventions were more likely to incur high expenditures, reinforcing the cost-intensive nature of operative management in emergency settings. The demographic profile of patients-predominantly young adults aged 21-40-aligns with the active working-age population, making the financial impact of OOP costs even more critical [11]. Facial injuries, the most frequent indication for plastic surgical involvement, were independently associated with higher costs, possibly due to the complexity and need for precision in cosmetic and functional repair. Furthermore, more than 48% of patients reported severe financial distress, emphasizing the psychological and economic consequences of unanticipated health expenses [12]. Lack of financial counselling and poor awareness of potential charges exacerbated the distress, with a majority of patients expressing a desire for upfront cost estimation and support [13]. These results highlight how urgently policy-level measures like enhancing emergency insurance portability, increasing government coverage and putting financial navigators in ERs are needed [14]. The hidden burden that patients undergoing required plastic surgery face can be lessened by hospitals by incorporating cost transparency and patient-centered financial guidance into emergency treatment [15].

## Conclusion:

The out-of-pocket cost of emergency plastic surgery care is high, especially for patients without insurance. Surgical procedures, pharmaceuticals and consumables are the main sources of expenses. To reduce patient suffering and increase access to prompt care, insurance coverage must be strengthened and financial counselling must be incorporated into emergency situations.
